# Dietary Patterns among Smokers and Non-Smokers: Findings from the National Health and Nutritional Examination Survey (NHANES) 2017–2018

**DOI:** 10.3390/nu16132035

**Published:** 2024-06-27

**Authors:** Wenxue Lin, Hani A. Alfheeaid, Ibrahim Alasqah, Nada Alqarawi, Saad Abdullah Alotaibi, Fatmah Fahad Alribdi, Sulaiman Almutairi, Maria João Lima, Edite Teixeira-Lemos, António Raposo

**Affiliations:** 1 Department of Epidemiology and Biostatistics, College of Public Health, Temple University, Philadelphia, PA 19122, USA; 2 Department of Food Science and Human Nutrition, College of Agriculture and Food, Qassim University, Buraydah 51452, Saudi Arabia; h.alfheeaid@qu.edu.sa; 3 Department of Community, Psychiatric and Mental Health Nursing, College of Nursing, Qassim University, Buraydah 51452, Saudi Arabia; i.alasqah@qu.edu.sa (I.A.); n.alqarawi@qu.edu.sa (N.A.); 4 School of Health, University of New England, Armidale, NSW 2351, Australia; 5 Department of Public Health, College of Applied Medical Sciences, Qassim University, Buraydah 51452, Saudi Arabia; s.alrowes@qu.edu.sa; 6 Director of Model of Care, Qassim Health Cluster, Buraydah 52367, Saudi Arabia; falribdi@moh.gov.sa; 7 Department of Health Informatics, College of Applied Medical Sciences, Qassim University, Buraydah 51452, Saudi Arabia; ssmtiery@qu.edu.sa; 8 CERNAS Research Centre, Polytechnic University of Viseu, 3504-510 Viseu, Portugal; mjoaolima@esav.ipv.pt (M.J.L.); etlemos3@gmail.com (E.T.-L.); 9 CBIOS (Research Center for Biosciences and Health Technologies), Universidade Lusófona de Humanidades e Tecnologias, Campo Grande 376, 1749-024 Lisboa, Portugal

**Keywords:** behavior, diet, frozen meals, NHANES, tobacco, ultra-processed foods

## Abstract

Diet behavior and nutrition are critical for maintaining health and improving quality of life. Cigarette smoking remains the leading cause of preventable death in the United States. Poor dietary choices, such as excessively frequenting restaurants, consuming ready-to-eat foods from grocery stores, and ingesting ultra-processed foods (like frozen meals and pizzas), can adversely impact health. Despite this, research comparing dietary behaviors between smokers and non-smokers is limited. Using data from the National Health and Nutritional Examination Survey 2017–2018, we analyzed diet behavior based on smoking status. Our findings reveal that smokers had a significant increase (90%) in the frequency of consuming frozen meals/pizzas in the past 30 days compared to non-smokers (coefficient: 1.9; 95% CI: 1.4, 2.6; *p*-value < 0.001). Additionally, over 70% of participants, regardless of their smoking status, were unaware of MyPlate, a nutritional guide created by the United States Department of Agriculture (USDA) to encourage Americans to make healthier food choices. There is an urgent need to increase public awareness of MyPlate and promote a better understanding of healthy dietary behaviors.

## 1. Introduction

Despite the decline in the cigarette smoking prevalence to 11.5% in 2021 [1,2,3], it remains the leading cause of preventable diseases and deaths in the United States, responsible for at least 480,000 deaths annually [2]. Over 28.3 million smokers are at an elevated risk of smoking-related diseases, including liver cancer, coronary heart disease, lung cancer, pancreatic cancer, mouth and throat cancer, and type 2 diabetes [2,4].

The United States Food and Drug Administration (FDA) is committed to reducing the nicotine content (RNC) of and banning the menthol flavor in cigarettes. A few studies have demonstrated the benefits of RNC in reducing tobacco toxicant exposure, nicotine dependence, and improving smoking behaviors [5,6,7,8,9,10,11,12,13,14]. In contrast, menthol has been shown to initiate smoking, especially among youths, and its disproportionate use and associated health disparities in the Black community exacerbate nicotine addiction, decrease quitting attempts, and increase cessation difficulties [15,16,17,18,19,20,21]. 

Most research to date has investigated the health impact of smoking behavior, nicotine dependence, smoke exposure, and different tobacco products (combustible cigarettes, electronic cigarettes, cigars, etc.) on tobacco users. However, few studies have assessed other lifestyle factors, such as dietary behaviors, between smokers and non-smokers. 

Ultra-processed foods (UPFs), which have undergone multiple processing steps and contain various ingredients, including preservatives, flavors, sugars, salt, and fats, constitute over 50% of the total dietary energy intake in the United States [22,23]. Ready-to-heat products, such as pre-prepared pies, pasta, and pizza dishes, are classic examples of UPFs [22]. Most UPFs contain excessive calories, sugar, sodium, multiple chemicals, and synthetic ingredients that can potentially promote chronic disease development [24,25]. Furthermore, the high consumption of trans fats, saturated fats, and cholesterol in daily UPFs has been linked to cardiovascular disease, leading to increased morbidity and mortality [26,27]. The high accessibility, convenience, rapid availability for consumers, high profitability, and extended shelf life for manufacturers contribute to UPFs’ popularity not only in the United States but also in Western countries, including the United Kingdom [28]. However, the high amounts of sugar, sodium, unhealthy fats, and nutritional emptiness [24] of UPFs are linked with multiple diseases, including cardiovascular disease [29], type 2 diabetes [30], and cancer [31]. For example, calorically dense frozen pizzas digest into glucose, leading to rapid spikes in glucose and insulin levels, which can contribute to tumor growth and diabetes [24]. 

A low socioeconomic status (SES) is significantly associated with a higher prevalence of smoking. Over 41% (95% CI: 39.8%–42.5%) of men with incomes below the federal poverty level smoke cigarettes, compared to 23.7% (95% CI: 23.2%–24.2%; *p*-value: <0.001) of men with incomes above the federal poverty level [32]. A similar association is found in women, with higher smoking rates in low SES women (32.5%; 95% CI: 31.4%–33.6%) compared to those with high SES (18.3%; 95% CI: 17.8%–18.7%; *p*-value: <0.001) [32]. Additionally, SES is related to UPF consumption. Low-income neighborhoods, where people have limited access to healthier food due to financial constraints, show a higher consumption of UPFs, such as frozen meals and pizzas [23]. 

Furthermore, certain populations of SES, who live in a congregated housing, such as college students, were observed to have a high prevalence of tobacco smoking [33]. Students tend to be exposed to unhealthy food choices, such as unhealthy snacking and foods with high calories as well as tobacco smoking practices [34,35].

Cigarette smokers living in poverty face greater health disparities due to exposure to nicotine and toxic chemicals [36]. Unhealthy dietary behaviors, such as frequent consumption of ready-to-eat foods or UPFs, can further exacerbate the health issues of tobacco users. However, few studies have investigated the differences in dietary behavior by smoking status. The FDA’s tobacco control policies mainly focus on reducing nicotine content to address addiction and flavor restrictions regarding initiation and cessation [37,38]. Healthy dietary behavior is another important factor to consider, as diet plays a critical role in health and disease prevention. In 2011, the U.S. Department of Agriculture (USDA) created MyPlate to provide nutrition guidance and recommendations based on the Dietary Guidelines for Americans [39]. The main purpose of MyPlate is to offer an individually tailored food plan to meet the participants’ needs and promote healthy eating habits [40]. However, the acceptance and awareness of MyPlate among the public remain unclear. Therefore, we aimed to compare the awareness of MyPlate and dietary behavior between smokers and non-smokers. 

## 2. Materials and Methods

### 2.1. The NHANES 2017–2018 Cohort

We used data from the National Health and Nutrition Examination Survey (NHANES) 2017–2018. The NHANES program, initiated in the 1960s, has been implemented across multiple surveys [41]. The NHANES is overseen by the National Center for Health Statistics (NCHS) and the Centers for Disease control and Prevention (CDC), providing a nationally representative survey of the non-institutionalized civilian US population [41]. A complex, multistage sampling procedure was used to generate nationally representative samples of the US population [42]. 

**Figure 1:**
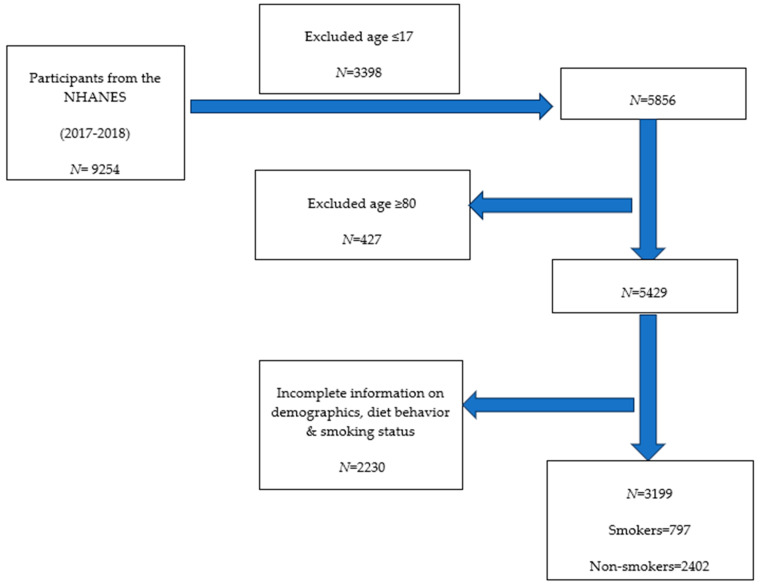
Flowchart of participants included in the study.

For the current study, we focused on individuals aged 18 years and older who provided complete data regarding demographic, dietary habits, nutrition, and cigarette smoking behaviors. From the initial 9254 participants in the NHANES 2017–2018 study, we excluded participants aged 17 years or younger (*N* = 3398) and those aged 80 years or older (*N* = 427), as well as those with incomplete information on demographics, diet behavior and nutrition (including number of meals not home-prepared and consumption of frozen meals/pizzas in the past 30 days), and smoking status (*N* = 2230). A total of 3199 participants met the eligibility criteria for our analysis, comparing diet behaviors and awareness of the MyPlate program between smokers and non-nonsmokers. Among the eligible participants, 23.8% (weighted proportion; unweighted *n* = 797) were identified as smokers, while 76.2% (weighted proportion; unweighted *n* = 2402) were non-smokers. Figure 1. shows the flowchart of participants included in the study.

### 2.2. Measures

The participants were dichotomized into smokers and non-smokers, based on their smoking status. Non-smokers were defined as individuals who had smoked fewer than 100 cigarettes in their lifetime, while current cigarette smokers were defined as those who had smoked at least 100 cigarettes in their lifetime and currently smoke cigarettes [43,44,45,46]. 

Demographic information on race/ethnicity (Mexican American, Other Hispanic, non-Hispanic White, non-Hispanic Black, and Others), education attainment (less than high school diploma vs. more than high school diploma, e.g., some college or college graduate or above), gender (male vs. female), and annual household income (USD 0 to USD 54,999 vs. more than or equal to USD 55,000) were collected using demographic data and classified as categorical variables. We kept body mass index (BMI, kg/m^2^) as both categorical and continuous variables in our study to facilitate the better comparison between smokers and non-smokers. Dietary behavior information was obtained from the Diet behavior and Nutrition (DBQ_J) questionnaire: (1) Number of meals not home-prepared: “By meal, I mean breakfast, lunch, and dinner. During the past 7 days, how many meals {did you} get that were prepared away from home in places such as restaurants, fast food places, food stands, grocery stores, or from vending machines?”; (2) Number of ready-to-eat foods in past 30 days: “Some grocery stores sell “ready to eat” foods such as salads, soups, chicken, sandwiches and cooked vegetables in their salad bars and deli counters. During the past 30 days, how often did {you} eat “ready to eat” foods from the grocery store?”; (3) Number of frozen meals/pizzas in past 30 days: “During the past 30 days, how often did you eat frozen meals or frozen pizzas?” Participants responded to these three questions as none (0 meals or 0 time), 1–21 meals for Question 1 (Number of meals not home prepared during the past 7 days), 1–90 times for Question 2 and 3 (Number of ready-to-eat foods in past 30 days and Number of frozen meals/pizzas in past 30 days, respectively). 

The awareness of MyPlate was assessed through the Diet Behavior and Nutrition questionnaire with the question: “Next, I’m going to ask a few questions about the nutritional guidelines recommended for Americans by the federal government. {Have you} heard of My Plate?”. The participants responded with “Yes” or “No”. We excluded invalid responses such as “Refused”, “Don’t know”, or missing.

### 2.3. Statistical Analysis

Unweighted counts, weighted means (for continuous variables) along with standard errors, and proportions (categorical variables) were provided. To compare participant characteristics based on smoking status, Rao–Scott chi-squared tests were used for categorical variables and *t*-tests were used for continuous variables [43,45,47,48]. We conducted a multivariable Poisson regression analysis to estimate the association between smoking status and diet behavior, accounting for count data in the response variables [49]. Both adjusted and unadjusted Poisson regression coefficients (exponentiated for easier interpretation) were examined and reported, along with the corresponding *p*-values and a 95% confidence interval (CI). We included demographic factors in our adjusted model to remove any potential confounding effects. 

Statistical analyses were performed using the R statistical software (version 4.3.3). The necessary R packages (such as survey packages and survey design) were loaded and used for statistical analysis, incorporating appropriate weights, strata, and clustering variables to accommodate the complex sampling design as recommended by NHANES [47,50,51,52,53]. All tests were two-sided, with the significance level set at 0.05.

## 3. Results

Table 1 presents the characteristics of the study participants, stratified by smoking status. Smokers had a younger age (43.2 vs. 45.8 years), higher proportion of male (54.4% vs. 40.8%), lower education attainment (less than high school: 54.3% vs. 30.7%), and a higher proportion of annual household income of lower than USD 55,000 (67.4% vs. 39.3%) than non-smokers (*p* < 0.05). They were also less likely to have heard of MyPlate (21.0%vs. 29.7%) and had a lower ratio of family income to poverty (2.2 vs. 3.3) compared to non-smokers (*p* < 0.05). However, there were no significant differences in race/ethnicity between participants based on smoking status (*p* > 0.05).

Table 2 presents the Poisson regression analysis examining the relationship between diet behavior and smoking status. In model 1, the mean frequency of consuming meals not home-prepared for smokers decreased by a factor of 0.9 (a 10% decrease) and 0.8 (a 20% decrease) in the crude and adjusted models compared to non-smokers, respectively; however, both associations were non-significant (*p*-value > 0.1). 

In model 2 (Table 2), the mean frequency of having ready-to-eat foods in the past 30 days for smokers increased by a factor of 1.2 (a 20% increase) in both the crude and adjusted models, with a non-significant association (*p* > 0.1) than non-smokers. 

In model 3 (Table 2), the mean frequency of having frozen meals/pizzas in the past 30 days for smokers significantly increased by a factor of 1.9 (a 90% increase; 95% CI: 1.4, 2.6; *p*-value < 0.001) and 1.7 (a 70% increase; 95% CI: 1.8, 2.8; *p*-value: 0.03) in the crude and adjusted models compared to non-smokers.

Overall, the frequencies of consuming frozen meals and pizzas in the past 30 days were higher among smokers compared to non-smokers. However, there were no significant differences between smokers and non-smokers regarding the consumption of meals not prepared at home or ready-to-eat foods in the past 30 days. Frozen meals and pizzas are usually popular due to their convenience and low economic cost, which may partially explain the higher frequency of use observed among smokers. 

## 4. Discussion

We found that there was no difference in the frequency of meals not home-prepared or ready-to-eat foods between smokers and non-smokers. However, the frequency of having frozen meals/pizzas (UPFs) was significantly higher among smokers than non-smokers. Although UPFs (e.g., frozen meals/pizzas) offer economic benefits with their affordability and convenience in preparation and access, they often contain excessive amounts of energy, sodium, and unhealthy fats, contributing to chronic diseases [29,30,54,55,56,57,58,59]. 

The FDA’s initiatives to reduce nicotine content (RNC) and ban menthol in cigarettes are essential steps in combating nicotine addiction and reducing smoking initiation, particularly among youths and marginalized communities. However, tobacco may not be the sole lifestyle factor that contributes to the adverse health outcomes observed in cigarette smokers. UPFs, prevalent in the American diet and characterized by their high sugar, sodium, and fat content, along with inadequate nutrient profiles [59,60,61,62], also pose significant health risks to the public. The frequent consumption of UPFs is associated with chronic diseases, such as cardiovascular disease, type 2 diabetes, and cancer [26,27,29,30,31], exacerbating health disparities, especially among smokers from low socioeconomic backgrounds. 

Of particular concern is the upward trend in UPF consumption among US adults from 2001–2002 to 2017–2018, with rates climbing from 53.5% to 57.0% [63]. Similarly, a steadily rising trend is observed in US youths, with the estimated percentage of total energy from UPFs consumption increasing from 61.4% to 67.0% from 1999 to 2018 [64]. This trend is particularly striking among non-Hispanic Black youths, where the consumption rate of UPFs surged from 62.2% to 72.5% [64]. 

Tobacco industries have aggressively marketed menthol cigarettes to Black individuals in urban communities [65], resulting in high menthol cigarette usage rates among Black youths and smokers [66]. Menthol has been linked to smoke initiation, especially among youths, and has been associated with lower success rates in quitting smoking compared to those of other racial/ethnic groups, exacerbating health disparities in the Black community [15,16,17,18,19,20,21,67]. The increased UPF consumption among Black youths may compound the negative health effects of menthol cigarette smoking. Based on the National Survey on Drug Use and Health, 85% of non-Hispanic Black, 29.4% of non-Hispanic White, 46.8% of Asian, and nearly 50% of Hispanic current cigarette smokers use menthol cigarettes [15]. The menthol content in commercially available cigarettes typically ranges from 1.61 to 4.38 mg per cigarette [68,69]. Tobacco manufacturers usually promote these products as menthol cigarettes. In addition to combustible cigarettes, menthol-flavored electronic cigarettes are also available for young adults, which poses another significant public health concern [70].

According to the Centers for Disease Control and Prevention (CDC), as of 2017–2018, over 30% of US adults were overweight, and 42.4% were obese (including 9.2% of severe obesity, defined as a BMI of 40 or higher) [71]. Obesity, weight gain [72,73,74], and UPF consumption are risk factors for type 2 diabetes [30], affecting over 30 million people in the US, with approximately 98 million suffering from prediabetes [75]. While various factors contribute to the health disparities observed in type 2 diabetes diagnosis [76], low socioeconomic status (SES), the overconsumption of UPFs, and smoking among racial and ethnic minorities and vulnerable populations play important roles in chronic disease development. The steadily increasing trend in UPF consumption among both US youths and adults poses significant public health challenges regardless of the smoking status. A similar increasing trend in UPF consumption has been observed in Korea, highlighting the global nature of unhealthy dietary behaviors [59]. Shim et al. investigated the Korea National Health and Nutrition Examination Survey 2010–2018, concluding an increased trend in UPF consumption from 23.1% to 26.1% across all socioeconomic statuses [59]. Therefore, unhealthy dietary behavior, including the frequent consumption of frozen meals/pizzas and other UPFs, is not only the public health concern in the US, Canada, and Western countries, but is also prevalent in Asia.

There is a notable correlation between smoking status and dietary habits, including the consumption and quantities of vegetables, fruits, and various types of diets [77]. Smokers have different nutrient and food intakes compared to non-smokers [77]. Specifically, non-smokers consume higher amounts of vitamin C, total fiber, and vitamin E across both genders [77]. Additionally, male smokers tend to consume less fruit, whole meal bread, and cereals but have higher overall energy intakes [77]. Furthermore, a study conducted by Norouzzadeh et al. found that current smokers with a poor diet quality—characterized by an inadequate consumption of vegetables, fruits, grains, fiber, and protein, along with a limited intake of food groups such as fish, eggs, and dairy—had a significantly higher risk of cardiovascular disease (CVD) and all-cause mortality compared to former smokers and light smokers [78]. This underscores the impact of smoking and dietary habits on overall health. 

The US Department of Agriculture (USDA) introduced MyPlate in 2011 to provide nutrition guidance based on the Dietary Guidelines for Americans [36]. However, our study found that only 21% of smokers were familiar with MyPlate, compared to 29.7% of non-smokers. In other words, more than 70% of participants had never heard of MyPlate, regardless of their smoking status. This low awareness rate, consistent with prior studies using NHANES 2013-2014 data [79], persists across various demographic groups, including college students, African American women, and older populations [80]. Despite being designed for adaptation by the US population to meet individual dietary needs, widespread exposure to MyPlate remains a challenge given its overall low awareness [81]. 

Multiple studies indicate that a poor diet and smoking contribute significantly to cardiovascular disease [82,83,84,85]. Plant-based dietary patterns show a strong evidence of reducing cardiovascular mortality in the population, regardless of smoking status [82]. In contrast, a high consumption of meats and sugar-sweetened beverages is particularly detrimental to health, especially among smokers [82]. Our findings reveal that smokers had a 90% increase in the frequency of consuming frozen meals and pizzas in the past 30 days compared to non-smokers, further suggesting the inadequacy of the diets observed among smokers. In addition, cigarette smoking is responsible for type 2 diabetes mellitus (DM). The correlations between diabetes/glucose tolerance decrease and cardiovascular disease, in conjunction with eating habits using NHANES data, could be further investigated in future studies.

Several limitations should be acknowledged. The prevalence of cigarette smoking has decreased to 11.5% due to anti-smoking campaigns, smoke-free laws, taxes, cessation tools, and improved perceptions of tobacco product risks, resulting in some former smokers. Our study did not differentiate between former and current smokers, which may impact the associations investigated regarding diet behavior and MyPlate awareness, as previous studies have shown that knowledge and risk perceptions vary by smoking status and use of different tobacco products [86,87,88]. In addition, electronic cigarettes (e-cigarettes) or Electronic Nicotine Delivery Systems (ENDS) are emerging as potential alternative tobacco products, particularly among youths, and may have different health effects compared to traditional cigarettes. Future studies comparing cigarette smokers and e-cigarette users regarding unhealthy diet behaviors or MyPlate awareness could provide valuable insights. Another notable limitation is the limited external validity. NHANES data are representative of residents within the US, limiting the generalizability of our findings to populations outside the United States [89]. Lastly, as a secondary analysis of publicly available data, our study is susceptible to potential bias from self-reported smoking status or the frequency of frozen meal/pizza consumption. 

However, our study also has strengths. NHANES provides a nationally representative sample, ensuring sufficient power for our analysis. To our knowledge, this may be the first study to comprehensively assess unhealthy diet behaviors between smokers and non-smokers, as well as MyPlate awareness by smoking status. While the US FDA, state health departments, and anti-smoking campaigns have made significant strides in tobacco control, as evidenced by the declining smoking rates, our study highlights the importance of addressing dietary behaviors that may jointly impact public health with cigarette smoking. We believe our findings may offer tobacco control policymakers a different perspective to implement more effective regulations regarding cessation and the assessment of the health effects of cigarette smoking and diet behaviors.

## 5. Conclusions

Our study reveals that smokers consume more frozen meals/pizzas (UPFs) compared to non-smokers. The rising trend of UPF consumption, especially among non-Hispanic Black youths, underscores the urgency of addressing dietary behaviors along with tobacco control efforts. The low awareness of MyPlate among smokers highlights the need for enhanced public health education. Future interventions should target both cigarette smoking and unhealthy diet behaviors to alleviate health disparities.

## Figures and Tables

**Table 1:** Characteristics of the participants by smoking status, 2017–2018 NHANES sample.

	Smokers *N* = 797 (23.8%)	Non-Smokers *N* = 2402 (76.2%)	*p*-Value
Gender			<0.001
Male	469 (54.4)	940 (40.8)	
Female	328 (45.6)	1462 (59.2)	
Race/ethnicity *			0.058
Mexican American	73 (7.0)	346 (9.4)	
Other Hispanic	40 (4.1)	247 (7.4)	
NH-White	354 (66.6)	656 (59.5)	
NH-Black	216 (12.3)	563 (12.3)	
Others	114 (10.0)	590 (11.5)	
Education			<0.001
<High school diploma	430 (54.3)	875 (30.7)	
≥High school diploma	367 (45.7)	1527 (69.3)	
Annual household income			<0.001
USD 0 to USD 54,999	602 (67.4)	1241 (39.3)	
≥USD 55,000	195 (32.6)	1161 (60.7)	
BMI (kg/m^2^)			0.084
Underweight (Below 18.5)	29 (3.2)	29 (1.5)	
Healthy Weight (18.5–24.9)	229 (29.3)	609 (25.5)	
Overweight (25.0–29.9)	231 (30.0)	771 (30.6)	
Obesity (30.0 and above)	308 (37.5)	993 (42.5)	
Heard of My Plate			
Yes	134 (21.0)	582 (29.7)	0.001
No	663 (79.0)	1820 (70.3)	
Age, years	43.2 (0.9)	45.8 (0.7)	0.02
BMI (kg/m^2^)	28.9 (0.4)	29.7 (0.3)	<0.001
Income to poverty ratio	2.2 (0.08)	3.3 (0.06)	0.02

Data source: NHANES 2017–2018. Categorical variables: unweighted *N* (weighted %). Continuous variables: *N* (mean). *p*-value was calculated by the Rao–Scott χ2 test and *t*-test for categorical variables and continuous variables, respectively. * NH: Non-Hispanic.

**Table 2:** Crude and adjusted Poisson regression models estimating the association between diet behavior and smoking status.

Model	Non-Smokers	Smokers Coefficient ^#^	95% CI	*p*-Value
(1) Number of meals not home-prepared				
Crude	Ref.	0.9	0.8, 1.0	0.11
Adjusted *	Ref.	0.8	0.7, 1.1	0.18
(2) Number of ready-to-eat foods in the past 30 days				
Crude	Ref.	1.2	0.9, 1.6	0.17
Adjusted *	Ref.	1.2	0.7, 2.1	0.3
(3) Number of frozen meals/pizzas in the past 30 days				
Crude	Ref.	1.9	1.4, 2.6	<0.001
Adjusted *	Ref.	1.7	1.8, 2.8	0.03

Data source: NHANES 2017–2018. ^#^ Coefficient from Poisson regression was exponentiated for interpretation. * Models adjusted for age, gender, race/ethnicity, education attainment, BMI, and annual household income.

## Data Availability

The NHANES data are publicly available on the CDC website: https://www.cdc.gov/nchs/nhanes/index.htm (accessed on 1 May 2024).
